# A Pulsed Electromagnetic Field Protects against Glutamate-Induced Excitotoxicity by Modulating the Endocannabinoid System in HT22 Cells

**DOI:** 10.3389/fnins.2017.00042

**Published:** 2017-02-06

**Authors:** Xin Li, Haoxiang Xu, Tao Lei, Yuefan Yang, Da Jing, Shuhui Dai, Peng Luo, Qiaoling Xu

**Affiliations:** ^1^Department of Anesthesiology, Xijing Hospital, Fourth Military Medical UniversityXi'an, China; ^2^Department of Biomedical Engineering, Fourth Military Medical UniversityXi'an, China; ^3^Department of Neurosurgery, Xijing Hospital, Fourth Military Medical UniversityXi'an, China; ^4^Department of Nursing, Fourth Military Medical UniversityXi'an, China

**Keywords:** pulsed electromagnetic field, excitotoxicity, endocannabinoid, glutamate, ERK

## Abstract

Glutamate-induced excitotoxicity is common in the pathogenesis of many neurological diseases. A pulsed electromagnetic field (PEMF) exerts therapeutic effects on the nervous system, but its specific mechanism associated with excitotoxicity is still unknown. We investigated the role of PEMF exposure in regulating glutamate-induced excitotoxicity through the endocannabinoid (eCB) system. PEMF exposure improved viability of HT22 cells after excitotoxicity and reduced lactate dehydrogenase release and cell death. An eCB receptor 1 (CB1R) specific inhibitor suppressed the protective effects of PEMF exposure, even though changes in CB1R expression were not observed. Elevation of N-arachidonylethanolamide (AEA) and 2-arachidonylglycerol (2-AG) following PEMF exposure indicated that the neuroprotective effects of PEMF were related to modulation of the eCB metabolic system. Furthermore, CB1R/ERK signaling was shown to be an important downstream pathway of PEMF in regulating excitotoxicity. These results suggest that PEMF exposure leads to neuroprotective effects against excitotoxicity by facilitating the eCB/CB1R/ERK signaling pathway. Therefore, PEMF may be a potential physical therapeutic technique for preventing and treating neurological diseases.

## Introduction

Neurological diseases are tightly associated with synaptic dysfunction involving an imbalance of excitatory and inhibitory transmitters. Overproduction of glutamate, which is the most important excitatory transmitter, in the synaptic cleft induces excitotoxicity and is the primary mechanism of acute brain injury, including stroke and brain trauma (Luo et al., 2011; Lai et al., 2014). However, clinical trials have reported unsatisfactory results for drugs designed to treat excitotoxicity (McConeghy et al., 2012). Neurosurgery can resolve mass effects caused by lesions, but it has no effect at the cellular level. Hence, novel therapies are required for treating excitotoxicity following brain injury.

Due to the closed cranial cavity and the blood–brain barrier, it is difficult to conduct traditional non-invasive treatments and develop drugs. A pulsed electromagnetic field (PEMF), as a non-invasive physical factor, can penetrate all tissues including skin, muscles, and bones (Yamaguchi-Sekino et al., 2011). Over the past decade, therapeutic effects of PEMF have been reported in a series of basic and clinical studies (Hug and Röösli, 2012). In the central nervous system (CNS), PEMF affects neurogenesis and neural recovery under normal or pathological conditions (Gramowski-Voß et al., 2015; Hei et al., 2016). Although the protective role of PEMF in stroke and brain trauma has been indicated, the biological mechanisms related to excitotoxicity at the cellular and molecular levels that could explain the therapeutic effects of PEMF have not been identified.

Endocannabinoids (eCBs), including N-arachidonylethanolamide (AEA) and 2-arachidonylglycerol (2-AG), are endogenous lipid mediators that act as key modulators of synaptic function (Tantimonaco et al., 2014; Parsons and Hurd, 2015). By activating cannabinoid receptors (CB1R and CB2R) expressed in the CNS, eCBs can regulate glutamatergic synaptic transmission and plasticity (Castillo et al., 2012). Since activation of eCB signaling inhibits excitotoxicity, it plays an important role in neuroprotection against ischemic/traumatic brain injury (Shohami et al., 2011; Benyó et al., 2016). In addition, previous studies showed that responsiveness of neural cells and tissues to physical stimuli such as electroacupuncture and transcranial magnetic stimulation was related to modulation of eCB signaling (Li et al., 2012; Wang H. N. et al., 2014). However, the biological effects of PEMF on neural eCB signaling is still unclear.

Hence, our objectives were to determine the biological effects of PEMF on excitotoxicity, clarify the relationship between PEMF and eCB signaling, and investigate the possible mechanism underlying PEMF regulation of excitotoxicity and eCB signaling. Through these studies, we hope to elucidate further the therapeutic effects of PEMF and its cellular mechanism in the CNS and to provide a potential strategy for physical therapy of neurological diseases.

## Materials and methods

### Cell culture

HT22 cells (a mouse hippocampal cell line) were obtained from the Institute of Biochemistry and Cell Biology, SIBS, CAS (Shanghai, China). The cells were grown in Dulbecco's Modified Eagle Medium (Thermo Scientific, Waltham, MA, USA) plus 10% fetal bovine serum (Hyclone Laboratories, Logan, UT) and 1% antibiotics (penicillin/streptomycin). One day before the experiments, cells were seeded in 6-well, 24-well, or 96-well culture dishes. Following treatment with glutamate and PEMF exposure, the cells were subjected to various measurements as described below.

### Antibodies and reagents

Primary antibodies to CB1R, DAGL, NAPE-PLD, MAGL, and FAAH were obtained from Cayman Chemical Inc. (Ann Arbor, MI, USA). Antibodies to p-ERK1/2, ERK1/2, and β-actin were obtained from Cell Signaling Technology (Beverly, MA, USA). The secondary antibodies for immunoblots were HRP-conjugated anti-rabbit, anti-mouse, and anti-goat IgG (Santa Cruz Biotechnology, Dallas, TX, USA). L-glutamate, ACEA, ACPA, AM251, AM281, 2-AG, AEA, PD98059, and U0126 were obtained from Tocris Bioscience (Avonmouth, Bristol, UK).

### PEMF treatment

The PEMF stimulation system was established as previously described (Wang J. et al., 2014). In brief, this system comprised a PEMF generator (GHY-III, FMMU, Xi'an, China; China Patent No. ZL02224739.4) and a solenoid surrounded with 400 turns of enamel-coated copper wire. A cell culture plate was placed in the center of the solenoid and received an open-circuit output wave form the PEMF at a frequency of 15 Hz. The peak-to-peak magnetic field was 9.6 Gauss. Measurement accuracy of the electromagnetic field output was confirmed using a gaussmeter (Model 455 DSP gaussmeter, Lake Shore Cryotronics). In the control group, each cell culture plate was placed in another solenoid that was also connected to the pulse generator but without an output waveform (no stimulation).

### Western blot analysis

After various treatments, HT22 cells in 6-cm dishes were washed three times with ice-cold PBS and lysed with lysis buffer containing protease inhibitor mixture tablets and phosphatase inhibitor mixture tablets (PhosSTOP, Roche Applied Science, Mannheim, Germany). Protein concentration of the supernatant was determined using a BCA protein kit (Beyotime, Shanghai, China). Proteins were separated by 10–15% and 10% SDS-PAGE gels and transferred to nitrocellulose membranes (Thermo Scientific). The membranes were soaked in 5% nonfat milk solution in Tris-buffered saline with 0.05% Tween 20 (TBST) for 1 h at room temperature and then incubated overnight at 4°C with the appropriate primary antibody (CB1, 1:800; DAGL, 1:800; NAPE-PLD, 1:500; MAGL, 1:500; FAAH, 1:800; p-ERK, 1:800; ERK, 1:1000; β-actin, 1:2500). Membranes were washed in TBST and incubated for 1 h at room temperature with the secondary antibodies diluted in blocking buffer. Immunoreactivity was detected by SuperSignal West Pico Chemiluminescent Substrate (Thermo Scientific). Optical densities of the bands were quantified using an image analysis system with ImageJ (Scion Corporation).

### eCB measurements

AEA and 2-AG were detected by LC-MS/MS (API 5000, AB SCIEX) as previously described (Valdeolivas et al., 2013). Briefly, cell lipid extracts were mixed with internal standards and analyzed by HPLC. After separation of AEA and 2-AG, MS analysis was conducted in the selected ion-monitoring mode. The m/z-values of 356 and 348 were used for deuterated and undeuterated AEA, while m/z-values of 384.35 and 379.35 were used for deuterated and undeuterated 2-AG. Calibration curves were produced using synthetic AEA and 2-AG. Analyst Software (version 1.4; Applied Biosystems) was used to evaluate concentrations of calibration standards, quality controls, and samples.

### Cell viability assay

Cell viability assays were performed using The Cell Proliferation Reagent WST-1 (Roche) following the manufacturer's protocol and culture cells (at a concentration of 0.5–5 × 10^4^) in microplates (tissue culture grade, 96 wells, flat bottom) at a volume of 100 μL/well. After treatment, 10 μL Cell Proliferation Reagent WST-1 was added to each well and incubated for 4 h at 37°C and 5% CO_2_. For background control (absorbance of culture medium plus WST-1 in the absence of cells), 100 μL culture medium and 10 μL Cell Proliferation Reagent WST-1 were added to a single well. Reaction mixtures were shaken thoroughly for 1 min on a shaker, and absorbance of the samples was measured against the background control blank in a microplate (ELISA) reader (Bio-Rad, Hercules, CA, USA).

### Lactate dehydrogenase (LDH) assay

Cytotoxicity was determined by measuring release of LDH, which is a cytoplasmic enzyme and a marker of membrane integrity. LDH release into the culture medium was detected using a diagnostic kit according to the manufacturer's instructions (Nanjing Jiancheng Bioengineering Institute, Nanjing, China). Briefly, 50 μL of supernatant from each well was collected to assay LDH release. The samples were incubated with the reduced form of nicotinamide-adenine dinucleotide (NADH) and pyruvate for 15 min at 37°C, and the reaction was stopped by adding 0.4 mol/L NaOH. Activity of LDH was calculated from absorbance at 440 nm, and background absorbance from culture medium that was not used for cell culture was subtracted from all absorbance measurements. The results were normalized to maximal LDH release, which was determined by treating control wells for 60 min with 1% Triton X-100 to lyse all cells.

### Statistical analysis

Statistical evaluation was done with GraphPad Prism software, version 5.0 (La Jolla, CA, USA). Significant differences between experiments were assessed by an ANOVA followed by Bonferroni *post-hoc* test (more than two groups) or unpaired *t*-test (two groups); *P* < 0.05 was considered significant. The data represent the values obtained from six to eight independent experiments, each of which was performed in duplicate.

## Results

### PEMF reduces glutamate-induced excitotoxicity in HT22 cells

**Figure 1:**
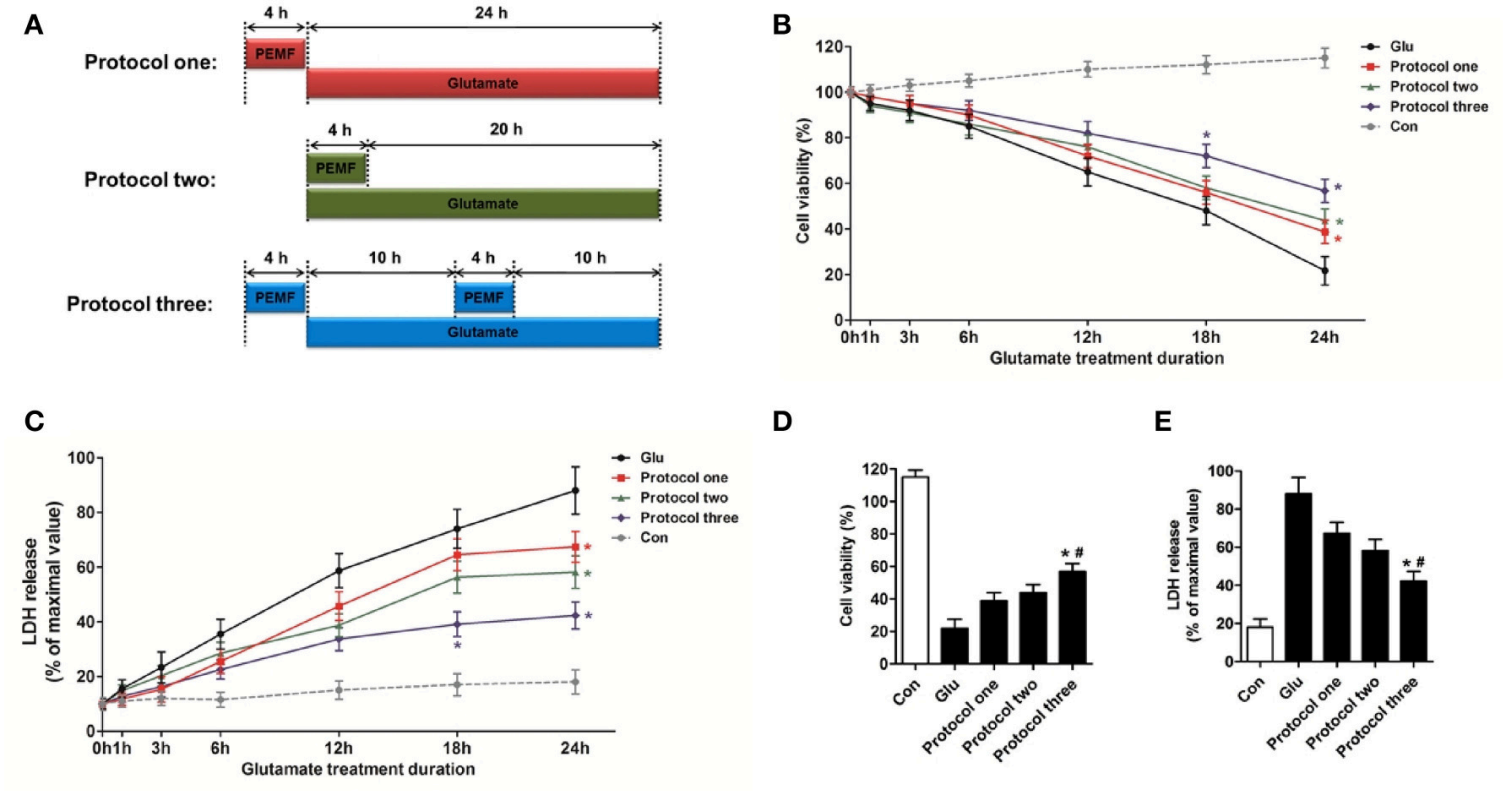
**Neuroprotective effects of PEMF against glutamate-induced excitotoxicity**. HT22 cells were treated with different PEMF and glutamate protocols as shown in **(A)**. Cell viability was measured by WST assay **(B)** and neurotoxicity was measured by LDH assay **(C)**. The data represent mean ± standard error from eight experiments. *F*-value **(B)** = 10.38, *F*-value **(C)** = 5.908, ^*^P < 0.05 vs. the glutamate (Glu) group. After glutamate treatment for 24 h, cell viability **(D)** and LDH release **(E)** were compared between the three protocols. The data represent mean ± standard error from eight experiments. *F*-value **(D)** = 48.82, *F*-value **(E)** = 18.83, ^*^P < 0.05 vs. protocol 1 group, ^#^*P* < 0.05 vs. protocol two group.

To investigate the biological effects of PEMF on glutamate-induced excitotoxicity, HT22 cells were treated with PEMF using three different protocols: (1) PEMF exposure for 4 h followed by glutamate treatment for 24 h; (2) PEMF exposure and glutamate treatment for 4 h followed by glutamate treatment for 20 h; (3) PEMF exposure for 4 h followed by glutamate treatment for 24 h with PEMF exposure for 4 h in the middle of glutamate treatment (Figure 1A).

After glutamate treatment for the indicated times (0, 1, 3, 6, 12, 18, and 24 h) under three different PEMF exposure protocols, viability of HT22 cells was higher than that of HT22 cells without PEMF treatment (Figure 1B), and PEMF exposure decreased LDH release after glutamate treatment (Figure 1C). Protocol 3 of PEMF exposure showed the most significant neuroprotective effects against excitotoxicity after glutamate treatment for 24 h (Figures 1D,E). Thus, this protocol was used for further experiments and defined as the PEMF + Glu group.

### Involvement of CB1R in PEMF-induced neuroprotective effects against excitotoxicity

**Figure 2:**
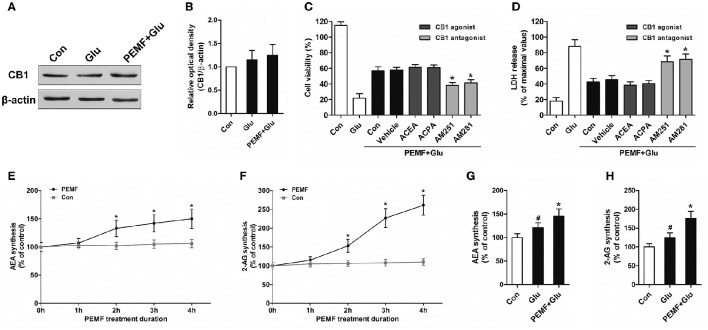
**PEMF exposure regulated eCB/CB1R signaling**. HT22 cells were treated with PEMF and glutamate according to protocol 3 for 24 h. Expression of CB1R was examined by western blot analysis **(A)**. The data represent mean ± standard error from 6 experiments **(B)**. After pretreatment with ACEA (30 μM), ACPA (30 μM), AM251 (10 μM), and AM281 (10 μM), cell viability **(C)** and neurotoxicity **(D)** were measured by WST and LDH assays, respectively. The data represent mean ± standard error from eight experiments. *F*-value **(C)** = 42.51, *F* value **(D)** = 14.27, ^*^P < 0.05 vs. vehicle group. After single PEMF exposure for indicated times, synthesis of AEA **(E)** and 2-AG **(F)** was assayed by LC-MS/MS. The data represent mean ± standard error from six experiments. *F*-value **(E)** = 9.823, *F*-value **(F)** = 10.56, ^*^P < 0.05 vs. control (Con) group. After treatment with PEMF and glutamate according to protocol 3 for 24 h, synthesis of AEA **(G)** and 2-AG **(H)** was assayed by LC-MS/MS. The data represent mean ± standard from six experiments. *F*-value **(G)** = 5.806, *F* value **(H)** = 7.496, ^#^*P* < 0.05 vs. control (Con) group. ^*^P < 0.05 vs. Glu group.

To clarify involvement of cannabinoid receptors in biological effects of PEMF, HT22 cells were treated with PEMF and glutamate. Expression of CB1R was not changed by PEMF in glutamate-treated HT22 cells (Figures 2A,B). Then, HT22 cells were pretreated with agonists of CB1R (ACEA and ACPA) and antagonists of CB1R (AM251 and AM281) before PEMF and glutamate treatments. Although activation of CB1R by ACEA and ACPA reduced glutamate-induced cell injury, these agonists did not affect the protective effects of PEMF against glutamate-induced excitotoxicity (Figures 2C,D). In contrast, AM251 and AM281, which inhibited CB1R, suppressed the PEMF-induced increase in cell viability and decrease in LDH release after glutamate treatment (Figures 2C,D).

### Neuroprotective effects of PEMF depend on elevation of 2-AG and AEA

To determine the role of PEMF in influencing production of eCBs, levels of two eCBs, 2-AG and AEA, were assessed in HT22 cells after treatment with PEMF for indicated times. Both 2-AG and AEA levels in HT22 cells were significantly elevated at 2, 3, and 4 h after PEMF treatment (Figures 2E,F). After glutamate treatment, either the 2-AG or AEA level was increased in HT22 cells under normal conditions or HT22 cells treated with PEMF (Figures 2G,H). However, levels of these eCBs in PEMF-treated cells were significantly higher than those of cells under normal conditions (Figures 2G,H).

### PEMF affects the eCB-related enzymatic system

**Figure 3:**
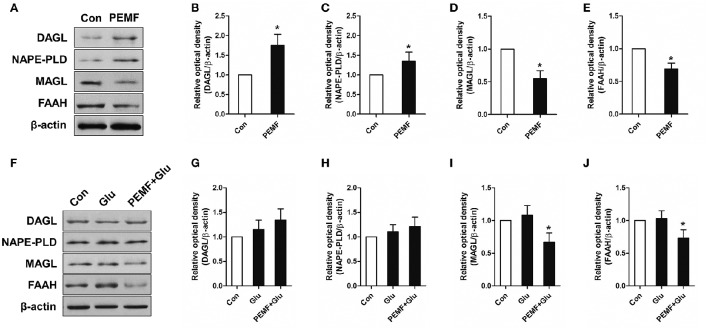
**Effects of PEMF exposure on expression of the eCB enzymatic system**. After single PEMF exposure for 4 h, expression of DAGL, NAPE-PLD, MAGL, and FAAH were examined by western blot analysis **(A)**. The data represent mean standard ± error from six experiments **(B–E)**. ^*^P < 0.05 vs. Con group. After treatment with PEMF and glutamate according to protocol 3 for 24 h, expression of DAGL, NAPE-PLD, MAGL, and FAAH were examined by western blot analysis **(F)**. The data represent mean ± standard error from six experiments **(G–J)**. *F*-value **(I)** = 7.365, *F*-value **(J)** = 5.563, ^*^P < 0.05 vs. Glu group.

To investigate involvement of the eCB-related enzymatic system in PEMF-induced neuroprotective effects, expression of diacylglycerol lipase (DAGL, an enzyme involved in producing 2-AG), monoacylglycerol lipase (MAGL, an enzyme involved in degrading 2-AG), N-acyl phosphatidylethanolamine phospholipase D (NAPE-PLD, an enzyme involved in producing AEA), and fatty acid amide hydrolase (FAAH, an enzyme involved in degrading AEA) were tested in HT22 cells under various conditions. After PEMF treatment, expression of DAGL and NAPE-PLD was increased, while expression of MAGL and FAAH was decreased in HT22 cells (Figures 3A–E). Although this elevation of DAGL and NAPE-PLD expression induced by PEMF was not significant after glutamate treatment, PEMF also reduced expression of MAGL and FAAH in HT22 cells undergoing excitotoxicity (Figures 3F–J).

### Activation of CB1R-ERK signaling contributes to the neuroprotective effects of PEMF

**Figure 4:**
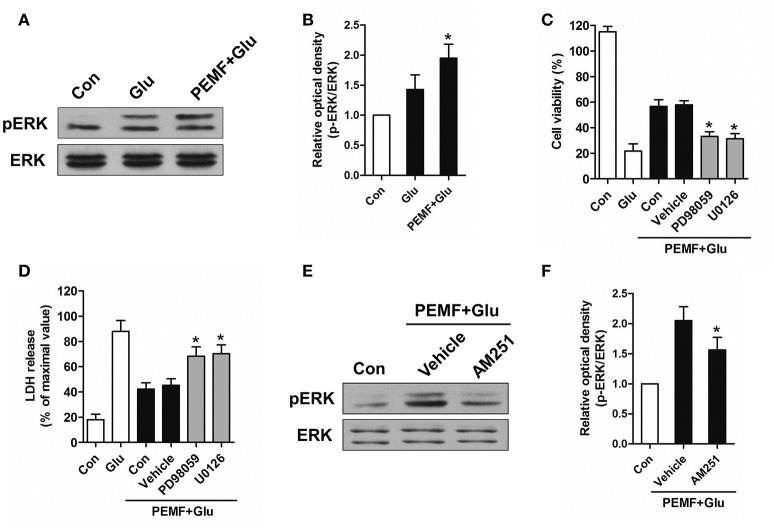
**Regulation of CB1R-ERK signaling by PEMF exposure**. HT22 cells were treated with PEMF and glutamate according to protocol 3 for 24 h. Expression of p-ERK1/2 and ERK1/2 was examined by western blot analysis **(A)**. The data represent mean ± standard error from six experiments **(B)**. *F*-value **(B)** = 6.144, ^*^P < 0.05 vs. Glu group. After pretreatment with PD98059 (40 μM) and U0126 (20 μM), cell viability **(C)** and neurotoxicity **(D)** were measured by WST and LDH assays, respectively. The data represent mean ± standard error from eight experiments. *F*-value **(C)** = 58.65, *F*-value **(D)** = 15.01, ^*^P < 0.05 vs. vehicle group. After pretreatment with AM251 (10 μM), expression of p-ERK1/2 and ERK1/2 was examined by western blot analysis **(E)**. The data represent mean ± standard error from six experiments **(F)**. *F*-value **(F)** = 8.537, ^*^P < 0.05 vs. vehicle group.

CB1R-ERK signaling has been implicated in regulating neuronal injury after excitotoxicity. To determine the role of CB1R-ERK signaling in neuroprotection induced by PEMF, HT22 cells were treated with PEMF. PEMF increased ERK1/2 phosphorylation in HT22 cells following glutamate treatment (Figures 4A,B). Pretreatment with ERK1/2 inhibitors, PD98059 or U0126, reduced the neuroprotective effects of PEMF against glutamate-induced excitotoxicity (Figures 4C,D). In the presence of AM251, an antagonist of CB1R, PEMF-induced phosphorylation of ERK1/2 was suppressed in glutamate-treated cells (Figures 4E,F).

## Discussion

The combination of low frequency magnetic fields and induction of microcurrents constitutes a basic mechanism underlying the biological effects of PEMF (Yamaguchi-Sekino et al., 2011). The benefits of PEMF have been evaluated by basic research and clinical trials in many countries. Application of PEMF exhibited therapeutic efficacy on bone fractures, skin ulcers, and lower back pain (Hei et al., 2016). Recent studies have shown that PEMF is a safe and non-invasive approach for management of several neurological diseases, including Alzheimer's disease (Arendash et al., 2010), Parkinson's disease (Vadalà et al., 2015), multiple sclerosis (Lappin et al., 2003), and brain injury (Rasouli et al., 2012; Pena-Philippides et al., 2014). However, the molecular mechanisms responsible for the therapeutic effects of PEMF on these diseases are still unclear.

Previous studies have shown that PEMF could modulate inflammation after traumatic brain injury by inhibiting production of pro-inflammatory factor IL-1β (Rasouli et al., 2012). Similar results were obtained from PEMF exposure after brain ischemia, which indicated that PEMF influences neuroinflammation via elevation of anti-inflammatory IL-10 and reduction of pro-apoptotic tumor necrosis factor (Pena-Philippides et al., 2014). In addition to inflammation, excitotoxicity induced by overproduction of excitatory transmitters, such as glutamate, participates in various pathophysiological processes of neurological diseases, especially acute brain injury (stroke and brain trauma) (Chamorro et al., 2016; Hinzman et al., 2016). In an attempt to reduce glutamate-induced excitotoxicity by PEMF at the cellular level, we showed that PEMF exposure could efficiently increase cell viability and inhibit LDH release in HT22 cells. This supports the hypothesis that glutamate-induced excitotoxicity is another target for the neuroprotective effects of PEMF against acute brain injury. In addition, we tried three different protocols for applying PEMF. Although a single PEMF exposure before (protocol 1) or after (protocol 2) glutamate treatment improved cell viability, a double PEMF exposure (protocol 3) showed better neuroprotective effects compared with a single PEMF exposure. This result suggests that multiple PEMF exposures with specific intervals might be a potential method for reduction of neuronal injury.

CB1R is highly expressed in glutamatergic synapses and can be activated by AEA and 2-AG. Since these two eCBs are produced by almost every neural cell, eCBs/CB1R signaling plays a significant role in regulating brain function (Xu and Chen, 2015). Considerable data support induction of this signaling system following exposure to physical treatments including magnetic and electric stimuli, which protect neural cells or the brain against various impact injuries (Wang et al., 2011; Mori et al., 2014). In this study, we found that CB1R could be a novel downstream molecule in the biological effects induced by PEMF. A CB1R inhibitor reduced neuroprotective effects due to PEMF exposure, even though PEMF exposure did not affect expression of CB1R. This indicates that PEMF might modulate activity of CB1R by regulating metabolism of eCBs, another important factor in the eCB system.

AEA was the first eCB isolated from the nervous system; it acts as an agonist in both the eCB and endovanilloid systems. Another major eCB, 2-AG, is specific for CB1R and CB2R and is present at higher concentration than AEA in the nervous system (Di Marzo et al., 2015). eCBs have been regarded as significant inhibitory mediators in synaptic excitation and have been shown to play neuroprotective roles in brain injuries by suppressing excitotoxicity (Xu and Chen, 2015). After confirming the relationship between PEMF and the eCB system as well as CB1R signaling, we found that levels of both AEA and 2-AG were elevated by PEMF exposure of cells under normal conditions. This effect induced by PEMF was also observed during glutamate-induced excitotoxicity. These results support the hypothesis based on previous experiments that PEMF modulates eCBs/CB1R signaling by influencing levels of eCBs and that the complex biosynthesis and degradation of eCBs is regulated by PEMF exposure.

Several enzymatic processes are involved in the metabolism of eCBs. AEA is synthesized from the hydrolysis of NAPE when catalyzed by NAPE-PLD enzyme. Production of 2-AG depends on the action of DAGL, which induces hydrolysis of DAGs. In contrast, FAAH contributes to the hydrolysis of AEA, while the hydrolysis of 2-AG is mediated by MAGL (Di Marzo et al., 2015). In this study, results suggested that PEMF exposure induced the elevation of NAPE-PLD and DAGL expression and reduction of FAAH and MAGL, leading to accumulation of eCBs. Although the effect of PEMF on changes in FAAH and DAGL during excitotoxicity was not significant, FAAH and MAGL were also regulated by PEMF exposure. Thus, we believe that the enzymatic system responsible for metabolism of eCBs is an important target of PEMF for neuroprotection. The different changes in enzymatic systems between cells under normal conditions and cells undergoing excitotoxicity might be related to the influence of glutamate on these enzymes, which counteracts the effects of PEMF exposure.

Activation of ERK signaling has been shown that induced the survival of neurons after glutamate-induced excitotoxicity (Ortuño-Sahagún et al., 2014). PEMF has been shown to activate ERK signaling in bone metabolism, but its role in regulation of ERK signaling in the CNS is not fully understood. The present study provides evidence that PEMF exposure promotes phosphorylation of ERK after excitotoxicity. Inhibition of this process by a specific ERK inhibitor reversed the neuroprotective effects of PEMF. In addition, activation of intracellular ERK signaling by CB1R is a crucial mechanism for neuroprotection (Kokona and Thermos, 2015). Further results showed that inactivation of CB1R could suppress PEMF-induced phosphorylation of ERK, indicating that PEMF exposure enhanced CB1R-ERK signaling. Taken together, we found that activation of ERK signaling was an essential downstream mechanism for PEMF-mediated regulation of the eCB/CB1R system and its neuroprotective effects against excitotoxicity.

There were several limitations in the present study. First, variation of stimulus conditions, including magnetic field intensity, frequency, duration, and time-varying mode, could change the biological effects of PEMF. Second, our results were obtained only from cellular experiments, which avoids influences from individual variation and complexity of animals or humans. Third, glutamate-induced excitotoxicity could not represent neurological diseases even though it is a crucial mechanism during pathogenesis of these diseases. Therefore, further studies of neurological effects of PEMF should be designed and conducted in animal models to elucidate its potential therapeutic effects and mechanisms. After ensuring the safety of PEMF, preclinical or clinical trials are needed to clarify its significance in clinical application.

## Author contributions

PL and QX conceived and designed the experiments; XL, HX, TL, YY, and SD performed the experiments; XL and PL analyzed the data; DJ and TL contributed reagents/materials/analysis tools; PL, XL, and QX wrote the paper.

## Funding

This work was supported by the National Natural Science Foundation of China (Nos. 81301037, 31270889, 81601149, and 81471806), and a China Postdoctoral Science Foundation funded project (No. 2015M572683).

### Conflict of interest statement

The authors declare that the research was conducted in the absence of any commercial or financial relationships that could be construed as a potential conflict of interest.

## Abbreviations

PEMF: pulsed electromagnetic field
eCB: endocannabinoid
CB1R: eCB receptor 1
CNS: central nervous system
AEA: N-arachidonylethanolamide
2-AG: 2-arachidonylglycerol
DAGL: diacylglycerol lipase
NAPE-PLD: N-acyl phosphatidylethanolamine phospholipase D
MAGL: monoacylglycerol lipase
FAAH: fatty acid amide hydrolase.
